# Chemotherapy-induced cavitating Wilms' tumor pulmonary metastasis: Active disease or scarring? A case report and literature review

**DOI:** 10.3389/fped.2023.1083168

**Published:** 2023-02-28

**Authors:** Angelo Zarfati, Cristina Martucci, Alessandro Crocoli, Annalisa Serra, Giorgio Persano, Alessandro Inserra

**Affiliations:** ^1^ General Surgery Unit, Department of Surgery, Bambino Gesù Children’s Hospital—IRCCS, Rome, Italy; ^2^ Department of Surgery, University of Rome Tor Vergata, Rome, Italy; ^3^ Surgical Oncology Unit, Department of Surgery, Bambino Gesù Children’s Hospital—IRCCS, Rome, Italy; ^4^ Hematology/Oncology Unit, Department of Pediatric Hematology/Oncology Cell and Gene Therapy, Bambino Gesù Children’s Hospital—IRCCS, Rome, Italy

**Keywords:** cavitation, atypical metastasis, nephroblastoma, Wilms’ tumor, pulmonary metastasis, lung metastasis, computed-tomography, chemotherapy

## Abstract

The second most common abdominal tumor in children is Wilms’ tumor, and the lung is where it most often metastasizes. The typical metastases are multiple, peripherally located, round, and variable-sized nodules. Atypical patterns are also possible and may create diagnostic challenges, especially in patients treated with chemotherapy. Among these, cavitating metastases are an anecdotal type of atypical secondary lung lesions. Here, we report a case of a chemotherapy-induced cavitating Wilms' tumor pulmonary metastasis discovered during the follow-up for an anaplastic nephroblastoma in a 6-year-old girl. Furthermore, we conducted a review of the existing literature on this exceedingly rare radiological pattern to establish its best management.

## Introduction

Wilms' tumor, the second most frequent extracranial malignant solid tumor in children, most commonly metastasizes to the lung (1). The most common radiologic appearance of lung metastases is multiple, spherical, and variable-sized nodules associated with diffuse interstitial thickening (2). However, atypical aspects of pulmonary localization of cancer may occur, and they could be more difficult to identify (3). Furthermore, treatments, as well as the biology of the tumor itself, may change the radiologic appearance of the metastasis and cause it to mimic other diseases, making a diagnosis difficult (2, 3). Early detection of pulmonary metastases in individuals with a known cancer may be essential for the design of a successful treatment plan (2). Only a few reports in the literature have described cavitation as an unusual evolution of pulmonary metastasis of the Wilms’ tumor (4).

Here, we report the case of a patient with known pulmonary metastasis secondary to Wilms' tumor in whom there was chemotherapy-induced cavitation of the metastasis itself and review the existing literature in this regard to establish the best management.

## Case description

A 4-year-old girl was referred to our institution for a palpable mass in the right quadrants of the abdomen; a CT scan revealed a localized Wilms' tumor arising from the right kidney. After initial staging, the patient was enrolled in the SIOP Umbrella 2016 protocol and started on a two-drug regimen (vincristine–actinomycin) for localized disease. After the first course, the patient experienced hemoperitoneum secondary to tumor rupture and underwent an urgent laparotomy and right nephrectomy. The histology confirmed the diagnosis of high-risk Wilms’ tumor III c, according to the UMBRELLA protocol, SIOP-RTSG 2016, with a blastemal predominance. The patient was started on adjuvant therapy according to a high-risk protocol (cyclophosphamide, doxorubicin, etoposide, and carboplatin) and whole abdomen irradiation (19.5 Gy, starting 30 days after surgery). She tolerated the treatment well and started the follow-up.

**Figure 1:**
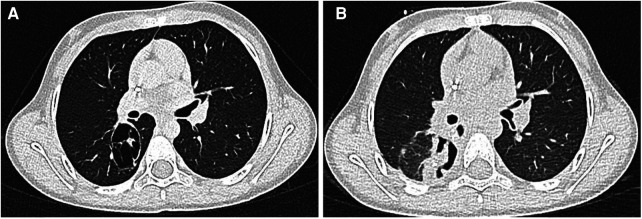
(**A,B**) At chest CT, a right 46 × 30 × 35 mm ovular, expansive pulmonary lesion and bilateral axillary lymphadenopathy was detected: the lesion showed non-uniform contrast enhancement.

**Figure 2:**
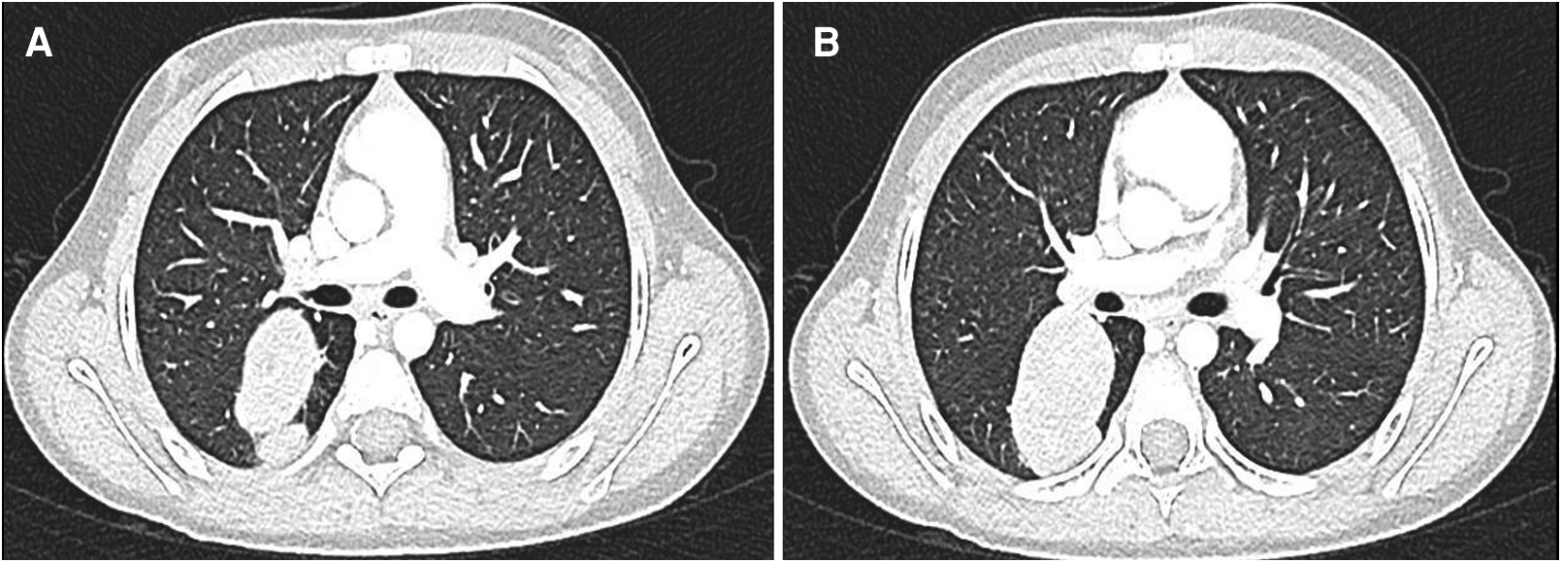
(**A,B**) After adjuvant therapy, a CT scan showed a persistency of the metastasis, which presented an unusual cavitating aspect.

During follow-up at 2 years after the initial diagnosis and 18 months after the last cycle of chemotherapy, a right ovular expansive pulmonary lesion and bilateral axillary lymphadenopathy were detected (Figure 1). The patient underwent preoperative chemotherapy with the vincristine (1.5 mg/m^2^)–irinotecan (50 mg/m^2^)–pazopanib (450 mg/m^2^) regimen. After the completion of three courses, a CT scan showed a persistency of the metastasis, which presented an unusual cavitating aspect (Figure 2). After consultations with the Institutional Tumor Board, the indication for surgical resection was established. She underwent segmentectomy of the superior segment of the right lower lobe by thoracotomy. No surgical complication occurred. The histology confirmed the diagnosis of Wilms’ tumor metastasis with a blastemal predominance, without any signs of anaplasia, and a residual vitality of 30%–40%. She was started on adjuvant therapies as per the UMBRELLA protocol with chemotherapy by following the vincristine/irinotecan/pazopanib regimen (nine postoperative courses) and lung irradiation (15 Gy from the 14th to 29th of June 2022). Two weeks after the surgery, a control CT did not rule out the disease’s persistence, and therefore, the treatment was continued. The patient is currently in adjuvant treatment and in good clinical condition. The main clinical events are summarized in Table 1.

**Table 1:** Time frame of the relevant clinical events.

February 22	February–April 22	April 22	May 22	May–October 22
CT detection of a right ovular expansive lung lesion during the second year of follow-up	Three courses of vincristine (1.5 mg/m^2^) – irinotecan (50 mg/m^2^) – pazopanib (450 mg/m^2^)	CT showed a persistency of the metastasis, which presented an unusual cavitating aspect	Segmentectomy of the superior segment of the right lower lobe by thoracotomy. The histology confirmed a viable metastasis	Nine courses of vincristine – irinotecan –pazopanib and lung irradiation (15 Gy)

## Discussion

We presented an exceptional case of chemotherapy-induced cavitation of a pulmonary metastasis of nephroblastoma. Wilms' tumor is the pediatric cancer most frequently associated with lung metastasis (3, 4). It usually appears as single or multiple, round, and well-defined nodules (4). Cavitation is an atypical presentation of Wilms’ lung metastases, rarely reported in the literature (5–8). In the literature, a pulmonary consolidation with a relatively thick wall (more than 4 mm) or within an adjacent infiltrate or mass that was detected during a radiological examination is referred to as a “cavity,” while a space containing air that is surrounded by a relatively thin wall (less than 4 mm) is referred to as a “cyst” (9). The causes of lung cavitary lesions cover a broad spectrum, from benign to malignant pulmonary disorders of congenital or acquired origin, as well as numerous infections (10). Due to the fact that they are typically created pathologically by necrotic tissue produced by an underlying lesion, cavity-forming pulmonary lesions are uncommon in the absence of a concurrent disease (9). Cavitation of malignancies may be caused by internal cyst formation, treatment-related necrosis, or internal desquamation of tumor cells followed by liquefaction (3, 9). Excavation of solid nodules with ejection of necrotic material within the tumor is the most likely etiology for cystic metastasis. Cystic dilation caused by a ball-valve obstruction in small bronchioles driven by tumor infiltration is another potential mechanism (3).

In the literature, only five cases of treatment-induced cavitation of pulmonary metastasis in patients affected by nephroblastoma have been reported (Table 2) (5–8). In all these patients, cavitation seemed to be induced by adjuvant chemotherapy associated with lung irradiation, while the presented patients did not undergo any lung irradiation before the cavitation appeared. Similarly to our patient, most cases of those reported in the literature had a single cavitating lesion, and a histological examination, when performed, always confirmed the diagnosis of a viable metastasis of Wilms’ tumor.

**Table 2:** Summary of cases reported in the literature.

Author, year	Cases	Chemotherapy	Chest radiotherapy	Number	Histology
Deck, 1959	1	Not reported	+ (30 Gy)	Multiple	Not performed
Coussement, 1973	1	+ (Actinomycin D)	+ (Dose not reported)	Single	Viable tumor
Kassner, 1976	2	+ (Actinomycin D, vincristine)	+ (10.5 Gy)	Single	Viable tumor, with no anaplasia and predominant sarcomatous component
		+ (Actinomycin D, vincristine)	+ (16 Gy)	Multiple	Not performed
Daneman 1978	1	+ (Actinomycin D, vincristine, adriamycin)	+ (19.5 Gy)	Multiple	Not performed

Controversy exists regarding the interpretation of the etiology and role of therapy-induced cavitating lesions. According to Seo et al., if the metastasis fails to shrink after appropriate treatment, it is usually constituted by a necrotic lesion (with or without fibrosis), lacking in live tumor cells (2). The only radiological difference between these “sterilized” nodules and a remnant live tumor seems to be the stable appearance of their size (2). Instead, according to Kassner, these alterations in metastatic lesions can be caused by tumor development rather than a side effect of treatment (8). This is in line with the findings in the present and the other reported cases, in which histologic examination invariably revealed a viable tumor in the pulmonary lesions (5, 6, 8).

Several preoperative factors influence the prognosis of patients with relapsed Wilms’ tumor with pulmonary metastases, including the persistence of pulmonary nodules after chemotherapy (16.7%–16.7% in 5-year-overall and event-free survival vs. 79.4%–66.5% in partial remission and 90.6%–79.4% in complete remission) and high-risk histology of the primary tumor (5-year-overall and event-free survival 44.4%–39.0% vs. 89.2%–75.9% in intermediate risk and 100%–93.3% in low risk, respectively) (11). According to the SIOP UMBRELLA 2016 protocol, the first treatment for relapsed metastatic disease is second-line chemotherapy; surgery for pulmonary metastases is indicated if a response to chemotherapy is apparent and when all persisting sites of the disease are amenable to complete excision (12). After local treatment, the presence of a viable tumor in the pulmonary nodules after chemotherapy and the persistence of lung metastases after local therapy (i.e., R1/R2 status after surgery or detectable metastases after radiotherapy) have also been associated with poorer survival in patients affected by metastatic and relapsed Wilms’ tumor (11). Patients who present with cavitating lesions on CT scan after chemotherapy, such as the patient in our case, pose a clinical challenge since the radiological appearance of pulmonary lesions in these patients does not reliably predict malignant behavior (13, 14). These patients need both histological confirmation of the vitality of the pulmonary metastases and complete resection of the residual disease to establish a subsequent treatment strategy and improve survival rates (11–13). Surgical removal of suspect lesions is, therefore, warranted (15).

## Conclusions

Chemotherapy-induced cavitating Wilms’ tumor pulmonary metastases are anecdotal atypical lung lesions that may create diagnostic challenges. In the presented case and in all cases previously reported, a histological exam confirmed the presence of a viable tumor in these lesions. Treatment-induced cavitating nephroblastoma lung metastases should be considered an active disease and removed for diagnostic and therapeutic purposes.

## Data Availability

The raw data supporting the conclusions of this article will be made available by the authors without undue reservation.
